# Women’s experiences of continuous support during childbirth: a meta-synthesis

**DOI:** 10.1186/s12884-018-1755-8

**Published:** 2018-05-15

**Authors:** Petronellah Lunda, Catharina Susanna Minnie, Petronella Benadé

**Affiliations:** NuMIQ Research Focus Area, Faculty of Health Sciences, North-West University, Private Bag X 6001, Potchefstroom, 2520 South Africa

**Keywords:** Birth companion, Childbirth experiences, Continuous childbirth support, Doula, Systematic review

## Abstract

**Background:**

Despite the known benefits of continuous support during childbirth, the practice is still not routinely implemented in all maternity settings and women’s views and experiences might not be considered.

The purpose of the study was to integrate individual studies’ findings related to women’s experiences of continuous support during childbirth in order to expand the understanding of the phenomenon. The review question was: What were the views and experiences of women regarding continuous support during childbirth as reported in studies that adopted qualitative or mixed research methods (with a qualitative component) using semi-structured, in-depth or focus group interviews or case studies?

**Methods:**

A detailed search was executed on electronic data bases: EBSCOhost: Medline, CINAHL, PsychINFO, SocINDEX, OAlster, Scopus, SciELO, Science Direct, PubMED and Google Scholar, using a predetermined search strategy. Reference lists of included studies were analysed to identify possible studies that were missing from electronic data bases.

Pre-determined inclusion and exclusion criteria were applied during the selection of eligible sources. After critical appraisal, a total of 12 studies were included for data-extraction and meta-synthesis.

**Results:**

Two themes were identified, namely the roles and attributes of the support persons and the type of support provided. Women’s perceptions about continuous support during childbirth were influenced by the characteristics and attributes of the support person as well as the types of supportive care rendered. Women preferred someone with whom they were familiar and comfortable.

**Conclusion:**

Continuous support during childbirth was valued by most women. Their perceptions were influenced by the type of support person: a health professional or a lay support person. Health care institutions should include continuous support during childbirth in their policies and guidelines.

**Electronic supplementary material:**

The online version of this article (10.1186/s12884-018-1755-8) contains supplementary material, which is available to authorized users.

## Background

Childbirth can be a life-changing experience for women, creating lifelong memories [1, 2]. Thus midwives should be familiar with women’s diverse needs during childbirth [3], including emotional, physical and informational needs [4]. Good interpersonal relationships could reduce fear associated with childbirth [5] and subsequently contribute to a satisfactory birth experience [6].

Continuous support during childbirth affects both the woman’s experiences and birth outcomes [4, 7]. It reduces the need for medical interventions, including medicated births, and improves both maternal and neonatal outcomes [4, 8, 9]. A review of continuous support for women during childbirth by Bohren et al. [4] found that through the provision of continuous labour support, various benefits were realised: ‘reduction in the need for epidural, assisted deliveries, caesarean section births, postpartum depression and neonatal admissions’. Similarly Pascali-Bonaro [2] reported; a reduction in the use of oxytocin, analgesia, instrument deliveries and duration of labour as well as improved maternal satisfaction, bonding with the baby and improved neonatal outcomes and reduced maternal anxiety [9].

However, irrespective of all the benefits, continuous childbirth support is not universally implemented probably due to a high rate of utilising medical interventions such as epidural anaesthesia in hospital settings [10]. Advances in medical technology during the last three decades contributed to an increased use of invasive procedures during childbirth [11]. Consequently, midwives might spend more time attending to technology and routine interventions than offering continuous support during childbirth [12]. In the absence of continuous support, women might feel deserted, distraught and petrified [13, 14]. Another factor that has been linked to the lack of continuous childbirth support is the shortage of midwives [15]. Where midwives are unable to render continuous support during childbirth, some aspects of care could be assigned to other support persons such as doulas, while midwives focus on the professional aspects [8].

The term midwife means ‘with woman’ [16]. Midwives therefore need to portray a caring and non-judgemental approach, have good communication skills, and be available to women [17–19]. The Royal College of Midwives [20], point out that a midwife has a role to guide women in making well-versed decisions that are acceptable clinically and personally. Hence, a ‘woman-centred’ approach is a necessity [15, 21–23]. The opinions and preferences of women should be heard and considered [15, 22, 23]. Considering women’s perceptions will generate information required for formulating guidelines pertaining to continuous support during childbirth. Therefore the undertaking of a meta-synthesis to establish women’s common perceptions is worthwhile.

Other associated meta-syntheses have synthesised women’s perceptions regarding varying aspects of childbirth. These meta-syntheses focussed on first time mothers’ experiences during early labour [24]; women’s experiences of caesarean births [25]; a secondary analysis of long-term memories and experiences of childbirth [1] and expert intra-partum care [26]. A similar meta-synthesis that reviewed and synthesised qualitative research evidence of women’s perceptions of professional labour support synthesised 17 qualitative studies [27]. However, the review focussed only on professional childbirth support, indicating the need for the current study.

The research question for the current study was: What is the best available research evidence about women’s views and experiences regarding continuous support during childbirth?

### Purpose

The purpose of the study was to integrate individual studies’ findings related to women’s views and experiences of continuous support during childbirth in order to expand the understanding of the phenomenon. A synthesis of findings of qualitative studies about women’s views and experiences, regarding continuous support during childbirth, will provide scientific evidence that is based on women’s views and experiences. More so a comprehensive body of knowledge about women’s views and experiences will highlight the plight of women on issues that are important to them. The synthesised summary will be availed to health care providers to utilise to render care that is acceptable to women depending on clinical needs and preferences.

## Methods

An explorative descriptive design, using a systematic review methodology, was followed to address the research question. Systematic review methodology incorporates a set of obvious, logical, structurally interrelated steps, carried out in a way that avoids bias and allows for peer review and independent verification [28].

The systematic review process followed five steps adapted from the guidelines for evidence analysis of the American Dietetic Association (ADA) [29]: (1) formulation of the review question and search strategy, (2) executing the search, (3) performing critical appraisal of selected studies, (4) summarising the evidence (data extraction from relevant studies and synthesising the findings) and (5) formulating the conclusion statements.

### Formulation of the review question and search strategy

The review question was formulated according to the SPIDER format. SPIDER is an efficient search strategy tool to use for qualitative and mixed research methods [30]. (Table 1).

**Table 1:** Elements of the review question according to the acronym SPIDER

Elements of spider	Elements of spider as applied to this study
S - Sample	Patients; mothers; women who had experienced childbirth
PI - Phenomenon of interest	Continuous support, intra-partum care; labour/labour support; companionship; doula; one-to-one care; emotional support
D - Design	Studies using qualitative and/or mixed research methods (with a qualitative component)
E - Evaluation	Experiences; perceptions; opinions; views
R - Research type	Semi-structured, in-depth or focus group interviews, case studies

Review question: What were the views and experiences of women regarding continuous support during childbirth as reported in studies that adopted qualitative and mixed research methods (with a qualitative component) using semi-structured, in-depth or focus group interviews or case studies to collect data?

The first author and the faculty librarian did a scoping search to assist in designing the search strategy. [31]. Focusing and periodic re-focusing enabled the identification of relevant studies [32].

The search was implemented using specific keywords based on the review question. The search words were used according to SPIDER (Table 1).

- women OR patients OR mothers AND
- experience* OR perception* OR opinion* OR view* AND
- continuous* AND
- labor* OR childbirth OR delivery AND
- emotional support OR intra-partum care* OR one-to-one care OR companionship* OR doula AND
- qualitative

The use of alternative words and spelling provides a more rigorous search thereby eliminating bias and the potential of missing relevant studies [33].

### Executing the search

The literature search comprised of an electronic and a manual search.

#### Electronic search

The following electronic databases: EBSCOhost, CINAHL, Medline, PsychINFO, SocINDEX, OAlster, Scopus, SciELO, PubMED were searched for studies reported in journals, dissertations and theses. SAePublications, Nexus and Google Scholar were also used to search for dissertations and theses not published yet (grey literature).

#### Manual search

Reference lists of included studies were scrutinised to identify any studies overlooked during the search from the databases [34, 35].

The search process was outlined using the PRISMA flow diagram [36]. (See Additional file 1: Figure S1: PRISMA flow chart).

Information of studies identified through the databases were exported and saved in RIS formatted folders and then imported into the EPPI-Reviewer 4 computer software [37]. It proved valuable to identify duplicate articles.

#### Eligibility assessment 

The next step was exclusion of irrelevant studies during screening of titles and abstracts that were deemed to be irrelevant to the research topic. Then full texts of potentially applicable studies were screened to determine whether or not they complied with the eligibility criteria [29, 38, 39]. (Table 2).

**Table 2:** Inclusion and exclusion criteria

Inclusion criteria	Exclusion criteria
• Studies related to women’s views, experiences and perceptions of continuous support during childbirth • Published between January 2005 and July 2016 • Written in English or with English abstracts • Primary studies	• Studies about continuous support not focusing on women’s experiences or views related to childbirth • Published before 2005 • Studies not written in English and without English abstracts • Not primary research • Non-research reports

### Critical appraisal of selected studies

Two team members independently appraised the papers for rigour and quality while the third member verified the decisions. The Critical Appraisal Skills Programme (CASP) tool for qualitative studies was used [40] because of its applicability to different qualitative study designs. The CASP tool assessed whether a study had a clear purpose and appropriate methodology, an appropriate research design to address the aims, a thorough description of the recruitment process and an appropriate data collection method, discussed data analysis, discussed the role of the researcher and reflexivity, addressed ethical issues, provided clear statements of findings and specified the implications for practice. Studies were appraised accordingly and the agreed upon cut-off score of 7/10 was applied ensuring that only rigorously executed studies were included in the synthesis. (Additional file 2: Table S1 Critical appraisal).

The critical appraisal revealed that most studies provided clear statements of the research aims, appropriate methodology, study design and data collection methods. They also provided sufficient information about the recruitment process, data analysis, findings, value of the research and contribution to practice. However, discussions of the role of the researcher and reflexivity and ethical issues were either insufficient or absent in most studies. Nonetheless only two studies [41, 42], were excluded as they failed to meet the cut-off score of 7/10.

### Data extraction

Once 12 relevant high quality studies had been selected, data extraction was done by the researcher and independently checked for relevance and correctness by a co-reviewer. A data extraction form was developed in such a way that its items answered the review question to ensure that no significant findings were omitted. It was then used as a guide to extract data from individual studies [29, 39]. (See Additional file 3: Table S2 Data extraction table).

### Data synthesis

As the synthesised findings of 12 primary studies were combined, the higher level synthesis is considered a meta-synthesis. The meta-synthesis involved identification and translation of similar concepts between the studies categorising women’s narratives into themes. The descriptive and analytical themes comprised the basis of the synthesis [33] to incorporate research evidence to answer the review question as outlined by ADA [29]. The synthesis was done using a thematic synthesis according to Thomas and Harden’s [33] guidelines. It involved combining data from 12 individual studies about women’s perceptions of continuous support during childbirth. The synthesis was done in three stages;

#### Stages one and two: Coding and developing descriptive themes

Coding of each text was done ‘line by line’ manually. Recurring concepts between studies were identified from women’s narratives and documented and then grouped according to similar meanings based on original accounts as descriptive themes. This process was repeated until no new concepts emerged from women’s narratives.

#### Stage three: Generating analytical themes

From the groups, two analytical themes that described women’s perceptions of continuous support during childbirth were generated with the remaining concepts as descriptive themes.

### Characteristics of the included studies

A total of 12 studies were analysed and included in the meta-synthesis. These studies included 651 women. Three studies had samples of primiparous women only [43–45], while the rest included both primiparous and multiparous women. The included studies described women’s views and experiences of childbirth support provided by different care givers in addition to midwives and nurses namely trained doulas, husbands, female relatives and friends.

Seven studies focused on women’s perceptions of general supportive care during childbirth [43, 45–50], while two studies reported on women’s experience of giving birth with their husbands’ support [44, 51]. The following themes were addressed in single studies: foreign-born women’s experiences of community-based doulas [52], women’s perceptions about labour companionship at public teaching hospitals [53], meaning and significance of a family member’s or friend’s support during childbirth [54]. Due to the different terms used to describe the investigated phenomenon in these studies, ‘perceptions’ was used inclusively throughout the findings’ discussion.

The birth settings in the individual studies comprised midwife-led units, hospitals and birth centres. The studies were conducted in Canada [45, 50, 54], Egypt, Lebanon and Syria [53], Malawi [43, 46], Nepal [44], Russia [51], Sweden [47, 49, 52], and the United States of America [48].

## Results

Quotations extracted from the original studies, are provided to support the themes. With regard to professional health workers, the term ‘midwife’ is used except when an individual study refers to nurses specifically.

The findings were synthesised under the analytical themes**;** the roles and attributes of the support persons, and types of support provided (See Additional file 4 Table S3: Theme and sub-themes).

### The roles and attributes of the support persons

Women’s perceptions were primarily influenced by their support persons.

### The support persons

The providers of supportive care comprised midwives, female relatives, friends or husbands [43–46, 48, 50, 51, 53, 54]. Others had community-based doulas (CBD) [47, 49, 52].

#### Female person

In general, a female support person was preferred because instinctively she knows and anticipates what another woman needs during childbirth [43, 46, 47, 50, 52, 53].

*“I really found my mom was a really big support throughout the whole thing…My mom’s gone through it three times.”* [45].

*“A mother is the best companion in labour…” She would be caring and I might forget the pain*… *“A woman feels what the other woman needs during labor and delivery, and knows how to deal with her*.” [53].

*“The family connection [her mother]…she’s known me my entire life. So, you know it’s somebody who I would feel very comfortable having around.”* [53].

A woman was highly appreciated for support as she understood another woman’s perspectives and has probably undergone a similar experience.

#### Midwives

The midwives worked collaboratively with women and their families [46, 50], enhancing the support persons’ confidence to perform their tasks. However, the midwives focused on the medical aspects of care hence, their supportive role was not apparent to the women [49, 52].

*“Mostly the medical things. No support at all [from midwives] I did not realize this since I totally relied on the doula.”* [49].

“*The Swedish midwives were there to check that the birth went well.”* [52].

In conclusion the decision to have a specific support person during childbirth depends on an individual woman’s preference. The basis for her preference has mitigating factors such as familiarity, previous experience or cultural background.

#### Doula

The doula provided care that was harmonising because she enabled the woman to ‘capitulate’ herself completely throughout the course of childbirth [49, 53]. One woman termed the doula as “a birthing sister” and “a positive witch.” [47].

*“Without her it would only have been him and me, we had only had each other and I can’t support him when I am giving birth, someone else has to do that.”* [47].

She was seen as the ‘missing piece in the puzzle’ [47] and provided care that was ‘intimate’ [49]. Foreign women found solace in the doula as someone they could relate to in their ‘own language’ in a ‘foreign land’ [52].

*“I had broken my arm and was very worried…I have no family in Sweden. I was wondering if I could do it, and the doula gave me a lot of support.”* [52].

Furthermore, doulas were the stronghold for the couples since husbands also needed emotional support during childbirth [45, 47].

#### Partner/husband

The husband offered ‘special support’ due to the intimate nature of the relationship [44, 45, 48, 54]. However, husbands took on a passive role when midwives took over during the second stage of childbirth [44].

*“My husband went away only when I was pushing. He preferred not to look. He was a little bit scared…maybe. I think it’s quite natural for men. It was fine because we discussed it before the delivery had started. I said he was free to do anything he likes. I understand that it could be quite stressful for him. Maybe even more stressful than for me, because I think we are designed for this much better* than men” [45].

*“You feel closer…we were always a really close family, but you feel even more of a bond…especially with your husband. You feel, wow, you’ve experienced this together.”* [54].

For some, the husband was the desired person to share the birth of the child with as he was part of the family unit [44, 45, 54].

*“His presence and positive affirmations sufficed. I felt like my husband took good care of me. He rubbed my back, and encouraged me to take frequent drinks to keep up the energy.”* [44].

Contrarily, some women were embarrassed or worried about their husbands’ presence as it was culturally unacceptable for husbands to witness their wives giving birth [44, 51, 53]. Like their Arab and Nepalese counterparts [44, 53], most Russian women [51] shared similar sentiments. They believed the birthing process does not require spectators, especially not husbands.

*“I could read his face. He was sweating and restless. It was very difficult for him to see me in pain. So my heart ached to see him suffer that way.”* [44].

*“This process is not for a man to see…it is repulsive and unsightly to see.”* [51].

*“I like my husband to be present but at the same time I am very shy…I don’t want my husband to see me in a situation where I am weak. It is also disgusting, so not nice for him to see you like this.”* [53].

Women’s perceptions of continuous support were influenced by factors such as the nature of the relationship, cultural orientation and gender. Women’s preferred support persons varied.

### Type of support provided

The type of support received and attributes of support persons, namely; physical presence, emotional support, physical support, information and advice, advocacy and interpersonal relationships also influenced their perceptions.

#### Physical presence

The continual presence of a support person provided a sense of security so it helped the woman to remain focused [48]. More so, a compassionate, caring, devoted and sociable support person was desired by women as they could express themselves freely [48, 53]. However, some women did not want bystanders during the actual birth process [54] for privacy and modesty [51].

*“…this is something a woman should go through alone” or that “this is the time to concentrate on yourself, and having someone else around will be a distraction.”* [53].

Favourably, the support person provided companionship [43, 49, 50, 52] so the women did not feel “gharib” (alone) [52].

*“I was very happy with my companion because at first I was afraid, I did not know what to do. You know people talk a lot. Someone told me that usually women are left alone without any nurse and sometimes they give birth while alone…”* [43]*.*

Evidently, the support person was able to provide a continuous presence due to the focus being on one woman [45, 48, 49, 52].

*“It was so wonderful to have her (doula) there with me, her just being there. There is something special about having her there…”* [49].

Though, midwives are the custodians of childbirth they could not provide continuous support due to their various roles [43, 49, 50], which resulted in women feeling neglected due to a discontinuous presence. Subsequently one woman compared the lack of constant support to a piece of merchandise waiting to be processed in a factory that does not need affection [49].

*“…Similarly when I had experienced something strange I asked her (companion) and she told me what to do*. *But there were no nurses*.” [43].

*“Nobody [midwife] came over and stroked my hair or just held my hand and said, you know, we’re coming right with you. So I was desperately hanging on to Ron [my partner].”* [50].

*“…the doula had her place, the midwife went in and out and was replaced. The doula was there all the time…”* [52].

In conclusion, most women appreciated the physical presence of a support person. More so, a lay person was highly cherished due the constant presence which a midwife could not provide because of multiple midwifery roles.

#### Emotional support

Most studies reported that women found emotional support to be inspiring [47, 52, 53]. Emotional support included affirmative words, reassurance, showing sensitivity to the woman’s fears, talking and praising for endurance, and providing spiritual inspiration [43, 46, 47, 52, 53].

“With the help of the doula I can trust my ability…she praised me when she heard how I handled my contractions; I could trust that I was on my way into the next stage. It was like an affirmation.” [47]*.*

*“I will have feelings of reassurance from the presence of a companion and from her encouragement.”* [53].

The emotional support received enhanced women’s self-trust and concurrently inner strength to bear the pain [44, 45, 49]. She was like a “real sister” because of her unwavering support and encouragement [54]. Positive affirmations from their support persons heightened women’s endurance to remain focused during childbirth. This was possible because the support persons were compassionate and caring.

#### Physical support

Comfort measures for relieving pain associated with childbirth included back massages, holding hands, and breathing techniques [43, 45, 49]. Mobilisation and adopting comfortable positions were also encouraged [43], birth balls were used for pelvic rocking [45]. Hygienic and elimination aspects were also attended to [43], enabling women to retain a state of physical well-being.

*“When I wanted to go to the toilet she held me by hand to and from the toilet, she made my bed so that I could sleep there comfortably. When the nurse said that I needed food because I was hungry, my mother prepared porridge for me to eat.”* [43].

*“Some people say the warm bath and stuff helps with the contractions...it didn’t help me and then we tried the shower, and I just found it irritating getting all wet and everything.”* [45].

*“I would hold my mom’s hand and squeeze her hand and she would say, “OK, you’re that much closer to getting to the end of the road...I’ve been through that contraction with you before”...That really helped a lot.”* [45].

Physical comfort measures facilitated relaxation and pain relief, though not for all women. Different techniques were applied depending on a woman’s needs and preferences. The support persons ensured that women’s physical needs were met.

#### Information and advice

Communication between the women and support persons heralded good interpersonal relations. The information and advice given was crucial for enhancing a woman’s childbirth experiences [43, 47, 48, 50, 52]. The support persons facilitated women’s self-awareness and confidence [47, 52].

*“My mother was advising me on what to do. She was advising me to lie on my sides, to turn, so I was listening to my mother. I was advised against screaming because it leads to exhaustion…”* [43]*.*

*“I did not know what happens, for example, when the baby comes out…I knew nothing about childbirth…The doula informed me about all these issues.”* [52].

*“It’s nice, ‘cause you just feel like quitting…to have three people [midwives and partner] encouraging you and cheering you on instead of it being just one.”* [54].

Provision of guidance during the early stages of childbirth resulted in women being more relaxed and cooperative [49, 52]. Women who are well informed of the indications for physical comfort measures would be more receptive to it. During active labour women were guided on positions to adopt as well as on breathing and relaxation techniques to expedite labour [43–45, 49].

*“She [doula] helped me to relax and use the oxygen, told me that I should not be afraid…My first child was born by caesarean section. This time it was 10 cm open, no complications…The doula was like a real sister and supported me so I wasn’t sad or angry.”* [52].

Consequently women had faith in the support person’s ability to provide relevant information and advice [47, 48, 52].

*“It was a supportive person who came along…one who had…been there before who knew. Not to help with the medical part but just to be there, support and explain what might happen, what you can ask for and so on.”* [47].

Preparation for childbirth should start during the prenatal period by educating and enlightening women about the birthing process and about accessible childbirth services to enable them to make informed decisions. Information from their support persons, in the absence of a midwife, provided relief and enlightenment.

#### Advocacy

Establishing communication channels with caregivers was desired by most women to ensure a common ground [47, 49, 50, 52]. Hence the support persons also represented the women when they could not speak for themselves [54].

An intermediary between the women and midwives or between women and family members, not present in the delivery room, was essential [54]. Since the doula was familiar with the birth environment, she acted as the intermediary [47, 48, 52, 54].

*“Security…knowing I was there with my sister…If I got to that point where I couldn’t speak up…My sister was my voice, and she knew me and knew my needs. So that gave me that sense of security that everything was going to be OK*.” [54].

*“I think they had the impression that they could come back in as soon as the baby was out, but…you don’t want people piling in when you’re being sutured…But at the same time, if someone could be a communicator for them because they have no way of finding out from us what was going on.”* [54].

Occasionally husbands also mediated, when there was less interaction from the midwives during the early stages of childbirth. Husbands were regarded as being more confident and flexible to convey the women’s needs to the midwives than female relatives [44]. Just prior to the ‘second stage’ of labour, some husbands interceded on behalf of their wives with the midwives [45] because women became apprehensive due to labour pains coupled with the unusual environment [47, 54].

*“He was determined to stay with me…he wasn’t there for the pushing but when the baby actually came out, he came back in. I needed my husband’s presence and his support.*” [45].

Due to the vulnerability of women during childbirth, their mediators ensured that their needs were met. The support persons played this role effectively. Through advocacy the spirit of togetherness was strengthened.

#### Interpersonal relationships

Some women preferred their husbands [44, 45, 54], female relatives or friends [46, 47, 50, 54] for support persons, based on the nature of their personal relationships as this permitted them to retain some control over the birth process. However, meeting the support person for the first time during childbirth caused women to feel apprehensive [46, 52]. A familiar support person enhanced the women’s feelings of security in the strange birth environment [53].

*“Our relationship was already great, but it just moved onto a totally different level because I really felt supported.”* [45].

*“For me it was very important to get to know the person who was going to be with me to feel secure with her before-hand.”* [49].

Compassion and openness towards women facilitated tranquility [45, 50]. More so, being acquainted with the support person earlier during pregnancy was cherished as it lead to good personal relationships and, in turn, a satisfactory childbirth experience [47, 52]. Women related more easily to persons with whom they shared beliefs than with strangers [46, 47, 52].

Good interpersonal relationships promoted women’s self-confidence and their trust in the support persons. Relatives and friends were complementary and represented close personal relationships. Even in the absence of a midwife, women never felt lonely. Consequently, good interpersonal relationships enhanced positive experiences.

## Discussion

The discussion of the current study’s findings were integrated with other literature investigating childbirth aspects such as women’s experiences of maternity services, the nature of modern midwifery practice, woman-centred care, doulas as support persons and companionship during childbirth.

Factors that influenced women’s perceptions regarding continuous support during childbirth will be discussed in relation to documents not included in the meta-synthesis. Implications of these studies’ findings will be addressed.

Women appreciated the continuous presence of support persons because of individualised attention [44, 45, 48, 49, 52]. Similarly Melender [55] found that the continual presence of a support person was reassuring and comforting. Jamas et al. [6], in a Brazilian study, revealed that one woman narrated feeling like ‘produce’ waiting to be ‘produced’ due to a lack of continuous support. The significance of continuous support during childbirth is endorsed by the World Health Organization (WHO) [56].

### The type of support person

The type of support persons varied in this meta-synthesis, including midwives, doulas, husbands and female relatives or friends. In North America doulas, trained as para-professionals [9, 45, 48], provide support, whereas in Arabian [44, 53] and African countries [43, 46], female relatives or friends with minimal or no training perform this function. Other studies [7–9] identified labour support providers, excluding midwives, as including female relatives or friends, husbands/partners, doulas.

The support person was expected to be gentle, empathetic and respectful towards the woman [45, 50], knowledgeable and culturally cognisant [5]. Other studies found that women expected a support person to be knowledgeable about childbirth, either through experience or training [42], for providing efficient and effective support.

### Female person

A woman was preferred for providing support as she was considered to be compassionate towards another woman [46, 47, 50, 51, 53]. These sentiments were echoed by Swedish women [47] who appreciated labour support from another woman deemed as being good at performing a “woman’s job.”

### Doula

In this meta-synthesis doula refers to a trained para-professional while support person includes all individuals that provide support. The current meta-synthesis indicated that the doula was perceived as the ideal support person compared to a relative or husband with vested emotions in the birth of ‘their baby’ [47, 49]. Doulas provided individualised attention to women [47–49], emotional support to husbands and were links between women and midwives [45–47].

Doulas provide care directly matching individual women’s needs and desires [48, 49, 52] by actively involving women in the birthing process and by empowering them through providing information and advice, as well as emotional and physical support [45, 48, 49, 52]. These findings are supported by the review of Bohen et al. [4] who identified the elements of childbirth support as ‘provision of information and advice, emotional support, continuous presence, comfort measures and advocacy’.

### Midwives

Midwives were unable to be continuously present [45, 49]. Unlike doulas, midwives perform multifaceted roles [45]. Supporting the findings of the current meta-synthesis, Byrom and Downe’s [57] findings highlight that a midwife provides comprehensive care to women but might be unable to provide individualised continuous support unlike lay support persons who can offer continuous support.

### Partner/husband

The father was favoured to be present during the birth of the baby by Canadian women [45, 53]. McGrath and Kennell [9] concur that the husband is an ideal support person because of the intimate relationship with the woman. However, for some women the husband’s presence was emotionally stressful [46] as husbands also needed support [42]. In some cultures, husbands are forbidden to witness childbirth [44, 51, 53].

Women’s preferred type of support persons was influenced by personal relationships, culture or birth setting. Lay support persons play a vital role which midwives cannot always fulfil due to their multiple roles. Conclusively support persons do not replace the midwife’s role but rather complement supportive care.

### Physical presence

The findings of the current meta-synthesis revealed that the presence of a support person was preferred as it facilitated the realisation of women’s needs and wishes. Women expected their support persons to be constantly present during the birthing process.

Women appreciated the continuous presence of support persons because of individualised attention [44, 45, 48, 49, 52]. Similarly Melender [55] found that the continuous presence of a support person was reassuring and comforting allowing women to be relaxed and calm.

However, the lack of continuous support from the midwives made one woman feel as though she was a piece of merchandise in a ‘production line’ waiting to be processed [49]. Similar Brazilian findings were reported by Jamas et al. [6] where one woman narrated feeling like a ‘produce’ waiting to be ‘produced’. Tanzanian [58] and South African [15] studies reported that women felt abandoned due to the lack of constant attention from midwives. Women’s reported sentiments highlight the value of having a support person whose sole purpose is to provide support.

### Emotional support

Another finding in this meta-synthesis was that emotional support was highly valued, as it enabled women to remain focused; consoled and gave them courage and strength to endure the process of childbirth [44, 45]. Emotional support comprised encouragement, empathy, and applause for fortitude [43, 45, 46]. Lundgren et al. [1] and Chan et al. [5] also found that gentle communication, praise, compassion and reassurance had calming effects on women. Furthermore, Lundgren et al. [1] and Pascali-Bonaro [2] highlight that childbirth has a lifelong impact on women. Thus, in order to heighten positive memories, support persons should be sympathetic towards women.

### Physical support

Physical comfort measures facilitated relaxation and provided pain relief. However, their use depended on a woman’s needs and preferences. They comprised massages, warm baths, breathing techniques and holding hands [43, 45, 49]. Assistance with mobilisation and adoption of more comfortable positions were also appreciated [44, 52]. These measures enabled women to go through the phases of childbirth in a state of optimal physical well-being. Green and Hotelling [59] and Simkin and Bolding [60] advocate for mobility and other comfort measures as a way of promoting and expediting spontaneous childbirth ensuring that women are not exposed to long periods of discomfort and painful labour.

### Information and advice

The current meta-synthesis showed that prenatal education enlightened women about the birthing process and accessible services enabling them to prepare for childbirth and make informed decisions. Information and advice from support persons fostered familiarity with the birthing process and practices [45, 47, 52] and enhanced women’s emotional stability [43, 47, 49, 52]. Bohren et al. [4] and Campbell [8] advocate that prenatal childbirth education and information during labour relieve anxiety enabling women to make informed decisions to improve both maternal and neonatal outcomes. Concurring with these findings is Melender [55] who found that women valued active involvement in decisions affecting them as it gave them a sense of self-worth unlike being mere recipients of instructions.

### Advocacy

Another revelation in this meta-synthesis was that, due to their vulnerability during childbirth, women need mediators to bridge the gap between themselves and midwives [44, 47, 54]. the support person present was such a ‘relay person’. Consequently, women felt assured and secure in the ‘unusual’ birth environment [45, 47, 49, 50, 52, 54]. These findings are supported by Melender [55] and Green and Hotelling [59] highlighting the need for a mediator during childbirth to convey women’s wishes to midwives and vice-versa. In this way women do not feel desolate. The relay of messages between women and midwives promoted a harmonious birth environment.

### Interpersonal relationships

The findings of this meta-synthesis show that good interpersonal relationships promote steadiness in the woman and trust in the support person, facilitating interactions with support persons. Women need to feel uninhibited during childbirth thus, some preferred their husbands as support persons because of the intimate relationship [44, 45, 54] as well as close female relatives or friends [46, 47, 50, 53, 54]. Kind-heartedness and sincerity from the support person facilitated the development of a trusting relationship with the woman [45, 50]. Pascali-Bonaro [2] reaffirms that trusting relationships between women and their support persons should be built timeously during pregnancy to avoid ‘unfamiliar encounters’ during childbirth.

## Conclusion

The findings of the current meta-synthesis demonstrated that women appreciated continuous support during childbirth. The influences were multidimensional and the benefits of continuous childbirth support were reaffirmed leading to the conclusion that continuous support during childbirth is an essential aspect of childbirth. However, women’s preference for specific support persons vary depending on interpersonal relationships, culture, values or birth environment.

Women prefer support persons with affirmative attributes to achieve positive outcomes. Health care institutions, policy makers, and professionals should recognise the significance of childbirth support. This should form part of antenatal education enabling women to make informed decisions based on the best available information. Consequently, women together with their preferred support persons, will be knowledgeable, adaptable and prepared for the actual childbirth experience. In situations of midwifery shortages, lay support persons provide a feasible and efficient alternative to providing continuous support during childbirth.

No South African studies were included as none met the selected inclusion criteria. Most analysed studies were from developed countries. Thus the context of the included studies might differ substantially from developing countries’ settings and might not be generalisable to developing countries. Some relevant studies might have been missed due to unclear titles or poor indexing.

## Additional files

Additional file 1: **Figure S1.** PRISMA Flow Chart. (DOCX 46 kb)

Additional file 2: **Table S1.** Critical appraisal. (DOCX 28 kb)

Additional file 3: **Table S2.** Data extraction. (DOCX 18 kb)

Additional file 4: **Table S3.** Themes and sub-themes. (DOCX 19 kb)

## Abbreviations

ADA: American Dietetic Association
CASP: Critical Appraisal Skills Programme
CBD: Community Based Doulas
CRD: Centre for Reviews and Dissemination
HREC: Human Research Ethics Committee
LDRP: Labour, Delivery, Recovery and Postpartum unit; NVD: Normal Vaginal Delivery
PRISMA: Preferred Reporting Items for Systematic Reviews and Meta-Analyses
RIS: Research Information Systems
SPIDER: Sample, Phenomenon of Interest, Design, Evaluation, Research type
WHO: World Health Organization
