# Supplementary material for: Women’s experiences of continuous support during childbirth: a meta-synthesis
Source: BMC Pregnancy Childbirth. 2018 May 15;18:167. doi: 10.1186/s12884-018-1755-8 (PMC5952857; doi:10.1186/s12884-018-1755-8)
Supplement: Supplementary file 1 — Figure S1. PRISMA Flow Chart. (DOCX 46 kb) [file 12884_2018_1755_MOESM1_ESM.docx]

**Identification**

Records identified through database searching

N=1560

Additional records identified through reference lists

N=7

Total number of hits through databases and extras through hand search

N=1567

Duplicates removed

N = 255

**Screening**

Records screened

N = 1312

(n=)

Records excluded: not relevant

N = 1283

**Eligibility**

E

Records screened on full text for eligibility

N = 29

Full-text articles excluded according to exclusion criteria:

- Not primary research = 2

- Non-qualitative element = 5

- Not women’s experiences of labour support = 8

- Did not meet cut-off point of 7/10 on critical appraisal =2

N=17

**Included**

Studies included in the synthesis

N= 12
