# Supplementary material for: Women’s experiences of continuous support during childbirth: a meta-synthesis
Source: BMC Pregnancy Childbirth. 2018 May 15;18:167. doi: 10.1186/s12884-018-1755-8 (PMC5952857; doi:10.1186/s12884-018-1755-8)
Supplement: Supplementary file 2 — Table S1. Critical appraisal. (DOCX 28 kb) [file 12884_2018_1755_MOESM2_ESM.docx]

| **Authors/title/ bibliographic information** | **Type of study design, setting and language of data collection** | **Sampling, sample size and demographics** | **Data collection technique and analysis** | **Rigour** |
| --- | --- | --- | --- | --- |
| Akhavan S, Edge D. Foreign-born women’s experiences of community-based doulas in Sweden-A qualitative study. Health Care Women Int. 2012; (33):833-848. | Design: Exploratory  Setting: hospitals in the Vastra Gotaland region, Sweden  Language: Arabic, Somali and Turkish. | Sampling: purposive  Sample: 10  Age: 23–31  Parity: 4 multiparous  Place of delivery: hospital  Mode of delivery: NVD  Post-delivery period: 3 and 6 weeks postpartum | Data collection: semi-structured interviews  Venue: women’s homes  Data analysis: content analysis | 1. Statement of aims of research: clear. 2. Methodology: appropriate and sufficiently discussed. 3. Research design: appropriate to address aims. 4. Recruitment: process sufficiently discussed. 5. Data collection method: appropriate. 6. Relationship between researcher and participants (reflexivity): Cannot tell. 7. Ethical issues: sufficiently considered. 8. Data analysis: sufficiently discussed. 9. Findings: a clear statement provided. 10. Value of research: discussed and its contribution to practice and the need for further research indicated.   C/A = 9/10  Included |
| Bakhta Y, Lee R. A survey of Russian women regarding the presence of a companion during labor. Int J Gynaecol Obstet. 2010; (109):201-203. | Design: qualitative survey  Setting: Omsk County  maternity and delivery hospital  Language: Russian | Sampling: convenient  Sample: 70  Age: 25.4 average  Parity: 1.6 average  Place of delivery: hospital  Post-delivery period 2 days post-partum (31 women) | Data collection: person-to-person interviews  Venue: hospital  Data analysis: content analysis | 1. Statement of aims: clear. 2. Methodology: appropriate and sufficiently discussed. 3. Research design: appropriate to address aims. 4. Recruitment process: sufficiently discussed. 5. Data collection method interviews were appropriate though women’s narratives are few. 6. Relationship between researcher and participants (reflexivity) sufficiently discussed regarding Russian speaking primary investigator. 7. Ethical issues: considered. 8. Data analysis: analysed by statistician. 9. A clear statement of findings was provided. 10. Value of research: discussed and its contribution to practice.   C/A=8/10  INCLUDED |
| Banda G, Kafulafula G, Nyirenda E, Taulo F, Kalilani L. Acceptability and experience of supportive companionship during childbirth in Malawi. BJOG 2010; (117):937-945. | Design: Cross-sectional surveys before and after introducing supportive companionship.  Setting: Maternity facilities in Blantyre City, Malawi.  Language: English and Chichewa | Sampling: variety of sampling methods  Sample: 220 first study and 192 second study  Age: ≤ 23-43 years  Parity: primiparous and multiparous  Place of delivery: hospital and health centers  Mode of delivery: NVD  Post-delivery period: while still in postnatal ward. | Data collection: mixed methods using interviews using questionnaire with closed and open-ended questions.  Venue: a private room at hospital  Data analysis: content analysis for qualitative data | 1. Statement of aims of research: clear. 2. Methodology: appropriate. 3. Research design: appropriate to address aims. 4. Recruitment process: sufficiently discussed. 5. Data collection: method interviews were appropriate to get women’s narratives though few. 6. Relationship between researcher and participants (reflexivity) not discussed. 7. Ethical issues: clear. 8. Data analysis, content analysis was sufficiently discussed. 9. A clear statement of findings was provided. 10. Value of research: Recommendations for further research were made.   C/A = 9/10  INCLUDED |
| Berg M, Terstad A. Swedish women’s experiences of doula support during childbirth. Midwifery. 2006; (22):330-338. | Design: Phenomenology  Setting: 2 large Swedish cities.  Language: Swedish and English | Sampling: purposeful  Sample:10 women (6 hospital delivery and 4 home)  Age: 25-35 years  Parity: primiparous and multiparous  Place of delivery: 6 in hospital and 4 at home  Mode of delivery: not specified  Post-delivery period: 1-2 months | Data collection: open-ended interviews 40-70 minutes  Venue: women’s homes  Data analysis: thematic analysis | 1. Statement of aims of research: is clear and relevant. 2. Methodology: appropriate. 3. Research design: appropriate to address aim. 4. Recruitment strategy: appropriate and selection process discussed appropriately. Reasons for size of sample given. 5. Data collection method was explicit and appropriate 6. Relationship between researcher and participants (reflexivity): not considered. 7. Ethical issues: considered sufficiently. 8. Data analysis: sufficiently discussed. 9. Findings: a clear statement provided. 10. Value of research: More research was recommended.   CA 9/10  INCLUDED |
| Etowa JB. Black women’s perceptions of supportive care during childbirth. Int J Childbirth Edu. 2012; 27(1):28-32. | Design: qualitative (not specified)  Setting: North End Community Health Centre located in the capital city of one of Canada’s Atlantic provinces  Language: English | Sampling : purposive  Sample:10  Age: 18-40 years  Parity: 5 primiparous and 5 multiparous  Place of delivery: healthcare institution  Mode of delivery: not specified  Post-delivery period: within 2 years and focus group interviews 6 weeks after interviews | Data collection: individual interviews and focus group interviews  Venue: not specified  Data analysis: thematic analysis | 1. Statement of aims of research: clear. 2. Methodology: is relevant and applicable in addressing the research goal. 3. Research design: only qualitative. 4. Recruitment strategy: appropriate to some extent, reason for sample size not given. 5. Data collection method: was appropriate, discussed to some extent. 6. Relationship between researcher and participants (reflexivity): not considered. 7. Ethical issues: considered to some extent. Rights of participants not discussed. 8. Data analysis: sufficiently discussed. 9. Findings: Only one key finding was discussed.   Value of research: No reference to further research made.  C/A = 6/10  EXCLUDED |
| Hunter C. Intimate space within institutionalized birth: Women’s experiences birthing with doulas. Anthropology Med. 2012; 19(3):315-326. | Design: Ethnography.  Setting: birth education centre in a mid-sized homogenous Midwestern town in America  Language: English | Sampling: purposive  Sample: 9 doulas and 9 mothers  Age: not specified  Parity: not specified  Place of delivery: hospital  Mode of delivery: 8 had NVD of which 2 used pain medication, 1 caesarean section | Data collection: observation during childbirth classes x 2 and observation in hospital. Doulas interviewed x 2 and mothers x1  Venue: birth education center  Data analysis: ethnographic analysis using Atlas ti qualitative software | 1. Statement of aims of research: clear. 2. Methodology: appropriate. 3. Research design: appropriate to address aims. 4. Recruitment strategy: snow ball technique was appropriate to address aim and process discussed. Reasons for size of sample given. 5. Data collection method: Data was collected over a long period and observations could be subjective. 6. Relationship between researcher and participants (Reflexivity): not discussed. 7. Ethical issues: considered. 8. Data analysis: sufficiently discussed. 9. Findings: a clear statement, provided. 10. Value of research: Further research suggested.   C/A = 8/10  INCLUDED |
| Kabakian-Khasholian T, El-Nemer A. Bashour H. Perceptions about labor companionship at public teaching hospitals in three Arab countries. Int J Gynaecol Obstet. 2015; (129):223-226. | Design: qualitative (not specified)  Setting: three large public teaching hospitals in Beirut (Lebanon), Damascus (Syria), and Mansoura (Egypt).  Language: Interviews in Arabic translated into English | Sampling: purposive  Sample: 69  Age: 18-38 years  Parity: primiparous and multiparous  Place of delivery: hospitals  Mode of delivery: NVD  Post-delivery period: immediate postpartum | Data collection: Semi-structured interviews  Venue: private space in the hospital  Data analysis: thematic analysis | 1. Statement of aims of research: is clear. 2. Methodology: appropriate. 3. Research design: appropriate to address aims. 4. Recruitment: purposive; process discussed. 5. Data collection method: appropriate . 6. Relationship between researcher and participants (Reflexivity): No proof. 7. Ethical issues considered. 8. Data analysis sufficiently discussed. 9. Findings: a clear statement provided. 10. Value of research: valuable as it contributes to practice.   CA 9/10  INCLUDED |
| Koumouitzes-Douvia J. Carr CA. Women's perceptions of their doula support. J Perinat Edu 2006; 15(4):34-40. | Design: only stated qualitative  Setting: hospitals  Language: English | Sampling: purposeful  Sample: 12  Age: 30-37 years  Parity: 11 primiparous and 1 multiparous  Place of delivery: hospital  Mode of delivery: 10 NVD, 2 caesarean section  Post-delivery period: 7 -16 weeks | Data collection: interviews  Venue: homes of participants  Data analysis: content analysis. | 1. Statement of aims of research: is clear. 2. Methodology: appropriate. 3. Research design: are appropriate to address aims. 4. Recruitment: the process was prone to biasness as the recruiters were the doulas. 5. Data collection method: appropriate, but not sufficiently discussed. 6. Relationship between researcher and participants (Reflexivity): not discussed. 7. Ethical issues: not addressed. Moreover subjects were paid. 8. Data analysis: sufficiently discussed. 9. Findings: a clear statement provided. 10. Value of research: Further research recommended.   CA 6/10  EXCLUDED |
| Kungwimba E, Malata A, Maluwa A, Chirwa E. Experiences of women with support they received from their birth companion during labour and delivery. Health. 2013; (5):45-52. | Design: descriptive  Setting: public hospital  Malawi  Language: Chichewa and English | Sampling: Purposive  Sample: 20  Age: 15-30 years  Parity: all primiparous  Place of delivery: hospital  Mode of delivery: NVD  Post-delivery period: immediate postpartum | Data collection: in-depth interviews using open-ended interview guide.  Venue: postnatal ward  Data analysis: content analysis-manually | 1. Statement of aims of research: is clear. 2. Methodology: appropriate. 3. Research design: are appropriate to address aims. 4. Recruitment: process discussed. 5. Data collection method: appropriate. 6. Relationship between researcher and participants (Reflexivity): Not discussed. 7. Ethical issues considered. 8. Data analysis sufficiently discussed. 9. Findings: a clear statement provided. 10. Value of the research: Contribution and implications for practice is discussed.   CA 9/10  INCLUDED |
| Lundgren I. Swedish women’s experiences of doula support during childbirth. Midwifery. 2010; (26):173–180. | Design: hermeneutic  Setting: 2 maternity hospitals in Sweden  Language: Swedish | Sampling: Purposive  Sample: 9  Age: 15-40 years  Parity: 7 primiparous and 2 multiparous  Place of delivery: hospital  Mode f delivery : 6 NVD, 1 planned, 2 emergency caesarean  Post-delivery period: 1-8 months | Data collection: interviews  Venue: woman’s home or place of woman’s choice  Data analysis: thematic analysis | 1. Statement of aims of research: clear. 2. Methodology: appropriate. 3. Research design: appropriate to address aims. 4. Recruitment strategy: purposive and process discussed. 5. Data collection method: appropriate. 6. Relationship between researcher and participants (Reflexivity): not discussed. 7. Ethical issues: considered to some extent. 8. Data analysis: sufficiently discussed. 9. Findings: a clear statement provided in relation to the research question. 10. Value of the research: contribution and implications for practice are discussed.   CA 9/10  INCLUDED |
| MacKinnon K, McIntyre M, Quance M. The meaning of the nurse’s presence during childbirth. JOGNN. 2005; (34):28-36. | Design: Hermeneutic phenomenological inquiry  Setting: urban center in Canada  Language: English | Sampling: purposive  Sample: 6  Age: Not younger than 36 years of age  Parity: 4 primiparous and 2 multiparous  Place of delivery: hospital  Mode of delivery type: NVD  Post-delivery period: 6 months | Data collection: open-ended interviews  Venue: not specified  Data analysis: thematic analysis | - Statement of aims of research: is clear. - Methodology: appropriate. - Research design: is appropriate to address aims. - Recruitment: purposive; process discussed. - Data collection method: appropriate. - Relationship between researcher and participants (Reflexivity): Not clear. - Ethical issues: not clear on permission, only that each woman was given a pseudonym. - Data analysis sufficiently discussed. - Findings: a clear statement provided. - Value of the research: implications for practice discussed and further research recommended.   CA 8/10  INCLUDED |
| Price S, Noseworthy J, Thornton J. Women’s experience with social presence during childbirth. Matern Child Nurs. 2007; 32(3):184-191. | Design: naturalistic  Setting: tertiary birth care  Eastern Canada  Language: English | Sampling: purposive  Sample: 16  Age: 22-37 years  Parity: 9 primiparous and 7 multiparous  Place of delivery: tertiary care birth unit  Mode of delivery: NVD  Post-delivery period: immediate postpartum before discharge | Data collection: open-ended interviews  Venue: postnatal ward  Data analysis: thematic analysis | 1. Statement of aims of research: is clear. 2. Methodology: appropriate. 3. Research design: are appropriate to address aims. 4. Recruitment: purposive; process discussed. 5. Data collection method: appropriate. 6. Relationship between researcher and participants (Reflexivity): Cannot tell. 7. Ethical issues considered. 8. Data analysis sufficiently discussed. 9. Findings: a clear statement provided. 10. Value of the research: implications for practice discussed.   CA 9/10  INCLUDED |
| Sapkota S, Kobayashi T, Takase M. Women’s experience of giving birth with their husband’s support in Nepal. J Brit Midwifery. 2011; 19(7):427-432. | Design: qualitative (not specified)  Setting: Midwife led birthing center located within the tertiary care maternity and women’s hospital in Kathmandu, Nepal.  Language: Nepali translated into English | Sampling: purposeful  Sample: 12  Age: 18 -28 years  Parity: primiparous  Place of delivery: birth centre  Mode of delivery: NVD  Post-delivery period: within 7 days | Data collection: in-depth semi-structured interviews  Venue: separate room within the birthing center  Data analysis: thematic analysis | 1. Statement of aims of research: is clear. 2. Methodology: appropriate. 3. Research design: not discussed, only mentions qualitative in abstract. 4. Recruitment: purposive; recruitment process discussed. 5. Data collection method: appropriate. 6. Relationship between researcher and participants (Reflexivity): discussed to some extent. 7. Ethical issues considered to some extent. 8. Data analysis: sufficiently discussed. 9. Findings: a clear statement provided. 10. Value of research: discussed and its contribution to practice and further research recommended.   C/A = 8/10  INCLUDED |
| Simpson MJ. Women’s perspectives on supportive care during labour and delivery. Vancouver: University of British Columbia (Dissertation-Master’s degree). 2012. | Design: interpretive descriptive  Setting: Labour, delivery, recovery and postpartum (LDRP) unit in a large metropolitan hospital  Language: English | Sampling: Theoretical snowball  Sample: 8  Age: 25-39  Parity: primiparous  Place of delivery: hospital  Mode of delivery :7 NVD (1 vacuum, 1 forceps), 1 caesarean section  Post-delivery period: 7 weeks – 7 months | Data collection: semi-structured, open ended interviews  Venue: women’s homes  Data analysis: thematic analysis | 1. Statement of aims of research: clear. 2. Methodology: appropriate. 3. Research design: appropriate to address aims. 4. Recruitment: purposive; recruitment process discussed. 5. Data collection method: appropriate. 6. Relationship between researcher and participants (Reflexivity): discussed. 7. Ethical issues considered. 8. Data analysis: sufficiently discussed. 9. Findings: a clear statement provided. 10. Value of research: conclusions contribute to existing knowledge and further research is recommended.   CA = 9/10  INCLUDED |
