# Supplementary material for: Women’s experiences of continuous support during childbirth: a meta-synthesis
Source: BMC Pregnancy Childbirth. 2018 May 15;18:167. doi: 10.1186/s12884-018-1755-8 (PMC5952857; doi:10.1186/s12884-018-1755-8)
Supplement: Supplementary file 3 — Table S2. Data extraction. (DOCX 18 kb) [file 12884_2018_1755_MOESM3_ESM.docx]

| **Author/**  **location** | **Methodology** | **Study focus sample** | **Sample size, parity** | **Birth**  **companions** | **Mode of delivery and place** | **Highlights from each study** |
| --- | --- | --- | --- | --- | --- | --- |
| Akhavan & Edge (2012) Sweden | Exploratory | Foreign born women’s experiences of community-based doula support | n=10 | Community- based doulas | NVD  Hospital | - Someone who speaks the same language in a foreign country, a desired companion, she is more than an interpreter. “I was so happy that someone who can speak both languages will follow me to hospital” - Motivated to become a doula once she has learnt Swedish - No continuity of support after childbirth-women were deserted soon after childbirth |
| Bakhta & Lee (2010) Russia | Qualitative survey | Attitudes of Russian women towards the presence of a support person during labor | n=70 | Husbands | NVD  Hospital | - Husband not ideal birth companion, should stay away from childbirth “if something goes wrong during labor, he will panic, faint, and hurt himself.” - No need to interfere in a natural process - Childbirth is a private moment no need for social interaction |
| Banda et al.(2010) Malawi | Variety of sampling methods | Women’s perceptions of companionship during labor | n=192 | Husbands, female relatives, mothers-in-law | NVD  Hospital  Health centre | - Companion very helpful, gave sound advice. ‘My companion was telling me what to do at different times’ - A companion is a mediator between woman and the midwives - A familiar female companion highly cherished |
| Berg & Terstad (2006) Sweden | Phenomenology | Women’s experiences of having a doula present during childbirth | n=10  5 primiparous  5 multiparous | Trained doulas | Hospital  Home | - The doula does it all; she is an all-rounder “She knows a lot about the childbirth. She has been there before. She can explain and clarify.” - She has a spectacular and phenomenal role - Doula a neutral person |
| Hunter (2012) United States of America | Ethnography | Mother-doula relationship during the birth experience. | n=9 | Trained doulas | 8 NVD  1 caesarean  Hospital | - The doula completes the childbirth process - “...but it was Henry [husband] and my doula that were supporting me. I don’t think it was like the nurses didn’t care about me...” |
| Kabakian-Khasholian et al. (2015) Lebanon, Syria and Egypt | Qualitative (not specified) | Perspectives of women, female relatives, and health care providers on labor companionship. | n=69  28 primiparous  41 multiparous | Female relatives | NVD  Hospital | - Female companion a very relevant person - Fear of labour pain and childbirth is equated to’ death’ and ‘re-birth’ - Husbands to witness the pain of childbirth in order to value wives. “He has to fear for my safety, he has to feel with me the pain I am going through and appreciate me for what I am going through.” |
| Kungwimba et al. (2013) Malawi | Descriptive | Participant’s knowledge regarding birth companionship. | n=20  Primiparous | Female relatives  Own mothers  Mothers-in-law | NVD  Hospital | - Some companions were unkind. “...surprisingly her response was that she was going home and will leave me alone. She left but came back after a nurse pleaded with her.” - Some birth companions gave contradictory advice from nurses - Given assistance during delivery by companion. “She was holding my head to raise me a little bit and assisted me to push effectively.” |
| Lundgren (2008) Sweden | Qualitative (not specified) | Women’s experiences of doula support during childbirth. | n=9  7 primiparous  2 multiparous | Trained doulas plus extra person; female friend, mother, partner | 6 NVD  3 caesarean  Hospital | - Birth is a life event focusing on the woman during antenatal and postpartum period - The doula mediated trust. “I heard her voice all the time and she calmed me down”. |
| MacKinnon et al. (2005) Canada | Hermeneutic | To develop a new understanding of what it means for a nurse to be present during childbirth. | n=6  4 primiparous  2 multiparous | Husbands, doula | NVD  hospital | - The husband and doula provide a dependable and valuable presence |
| Price et al. (2007) Canada | Naturalistic | The meaning and significance women attach to family and friend presence during childbirth within a tertiary care birth unit | n=16  9 primiparous  7 multiparous | Husbands/ partners, female relatives or friend plus extra person;  own mothers, or doula | NVD  tertiary care birth unit | - Husband the preferred choice for intimate family time and participant in childbirth. “ - Woman’s event, so she chooses companion - Comprehensive support - Childbirth is a personal and private moment |
| Sapkota et al. (2011) Nepal | Qualitative (not specified) | To explore women’s experiences of giving birth with their husbands’ support in urban Nepal. | n=12 primiparous | Husbands | NVD  Midwife-led birthing centre | - Embarrassment, discomfort and feelings of guilt by husband’s presence - Husband passive, not needed - For others, husband’s presence a source of comfort and calmness “I felt like my husband took good care of me. He rubbed my back, and encouraged me to take frequent drinks to keep up the energy.” |
| Simpson (2008) Canada | Interpretive descriptive | Women’s perceptions of the support that they received during labour and delivery. | n=8 primiparous | Husbands plus female person | NVD, forceps, vacuum, caesarean section  Hospital | - It is “ essentially mandatory for husbands to be present the birth of their babies” - Husband was wanted but not during the active phase before “pushing” |
