# Supplementary material for: Women’s experiences of continuous support during childbirth: a meta-synthesis
Source: BMC Pregnancy Childbirth. 2018 May 15;18:167. doi: 10.1186/s12884-018-1755-8 (PMC5952857; doi:10.1186/s12884-018-1755-8)
Supplement: Supplementary file 4 — Table S3. Themes and sub-themes. (DOCX 19 kb) [file 12884_2018_1755_MOESM4_ESM.docx]

| **Study** | **The type of support persons** | **Physical presence** | **Provider of emotional support** | **Provider of physical support** | **Provider of information and advice** | **Advocacy role** | **Interpersonal relationships** |
| --- | --- | --- | --- | --- | --- | --- | --- |
| Akhavan & Edge (2012) | Doulas   - The doula was a source of fulfillment represented “human rights and dignity” | - The doula eliminated the feeling of “gharib” (isolation) | - Softened the feeling of loneliness - Gained confidence - Fears conquered | - Provision of oxygen - Breathing techniques | - Doula knowledgeable and experienced - She gave the much needed advice | - Father-doula-midwife partnership | - Common language - Treated with respect and dignity - Doula-woman relationship established during antenatal period - Continued support during postnatal period |
| Bakhta & Lee (2010) | Husbands   - Husband’s presence was unacceptable due to cultural orientation - Childbirth is a private moment no need for spectators | - Private event no need for social interaction | ______ | ______ | ______ | ______ | ______ |
| Banda et al. (2010) | Female relatives or friends   - Women were pleased to have female relatives | - Acceptable companionship | - Sympathetic - Continual presence - Reassurance and praise | - Mobilisation - Food and water - Position changing - Elimination-both bowel and urinal | - Source of vital information through instructions | - Communication link between women and health professionals | - Witness to the birthing process - Provided companionship |
| Berg & Terstad (2006) | Doulas   - The doula was desired and comforting in her care - “A birthing sister” and “a positive witch” | - The missing “piece” of the “puzzle” - Accessible presence | - Readily available and dependable presence - Reassurance | - Massages, entonox, cold compresses to the forehead - Water to drink - Hygienic needs | - Extensive experience; explains and clarifies - Relevant advice | - A link between the couple and midwife   The doula a “guarantor” | - “Sisterly” relationship - “An affirmative person” |
| Hunter (2012) | Doulas and husbands   - The doula provided “womanly support” - Doula-husband partnership | - Great to have a doula present “Being with me” | - A caring and constant presence - Individualised attention | - Hand holding, stroking the hair, giving water, - Hygienic measures such as wiping of blood from legs and assistance with going to the bathroom | - ______ | - Represents one voice in labour | - Very close and personal with the doula - Familial relationship with husband |
| Lundgren (2008) | Doulas   - The doula respected the woman’s wishes   Husbands, female relatives | - The doula “helped me all the time” | - Prenatal doula support facilitated composure, it was “ like being in a bubble” - Praise and reassurance | - Entonox inhalation, massages and breathing techniques | - Boosting guidance - A teacher | - The doula a link between the couple and midwife - The doula was a source of security and trust | - The doula provided a sisterly presence - A personal relationship with the doula |
| Kabakian-Kasholian et al. (2015) | Female relatives   - Highly appreciated - “A woman knows how to deal with her” - “The support of your family gives you power.”   Husbands   - Husband’s presence was unacceptable by most women | - A support person mediates protection | - Another woman knows best - Reassuring and encouraging presence - “She would be caring and I might forget the pain” | - Holding of hands - “I would feel comforted when holding her hand” - Mobilisation | - “A companion should be well informed.” - Received advice on what to do during labour pains | - _____ | - Familial and trustworthy relationship - Reassuring and encouraging presence |
| Kungwimba et al. (2013) | Female relatives   - Female support person highly appreciated | - Support person mediates security | - Source of security - Reassurance and praise - Dependable and prayer partner | - Back massage, warm bath, food and fluids, changing of beddings, mobilisation and accompaniment to the toilet | - Provision of instructions during second stage - ”My mother was advising me on what to do....” | _____ | - Close relationship with “own mother” |
| MacKinnon et al. (2005) | Husbands and doulas   - Both were appreciated as they provided complementary support | - Reassuring presence complemented each other | - Completely present - Caring and not clinical | - Stroking of hair - Breathing techniques | - Knowledgeable and dependable - Continuous feedback - Skilled and competent | ______ | - Close relationship with husband - Doula complementary |
| Price et al. (2007) | Husbands and female relatives   - Both provided a sense of familiarity - “My birth… My choice” | - Aided them to “pass the time” or “forget the pain.” “Security…knowing I was there with my sister…” | - Continual presence - Compassionate and unconditional support - Comfort and control - Encouragement and reassurance | - “It was nice to have two people there, one person who was…holding me and another person reminded me to relax my shoulders or jaw or whatever” | - Expert advice - Provision of information about childbirth | - The link between woman and healthcare providers | - Intimate relationship with husband - Individualised support - Family bond and share in birth experience - “Security…knowing I was there with my sister…” |
| Sapkota et al. (2011) | Husbands and female relatives   - Husbands not favoured by most women - A female support person was preferred | - Mixed emotions prevailed regarding husbands’ presence | - Acknowledge-ment of efforts by husbands - Husbands’ presence empowering and a source of inner strength | - Provision of fluids - Position changing - Back rubs - Breathing and pushing techniques | - Guidance on physical measures to relieve pain | - Husbands were a communication link between wives and midwives | - Intimate relationship but no husband allowed during childbirth itself (pushing) |
| Simpson (2008) | Husbands and doulas   - Both provided highly valued support - There were reservations regarding husbands presence during pushing | - Both intermittent and continuous presence was acceptable - Husband’s presence “pivotal” | - Humour, empathy and kindness - Self-awareness and inner-control | - Back massages, visualisation, breathing and distraction techniques counter pressure, hydrotherapy - Self-hypnosis - Epidural | - Instructions on how to push during the second stage - Informed choice - Information on progress of labour | - Doula an advocate who stands up for women - Doula supporter for the primary support person | - Emotional connection with husbands - Shared intimate experience with husbands |
